# Development and validation of a novel preoperative clinical model for predicting lymph node metastasis in perihilar cholangiocarcinoma

**DOI:** 10.1186/s12885-024-12068-1

**Published:** 2024-03-04

**Authors:** Shuqi Mao, Yuying Shan, Xi Yu, Yong Yang, Shengdong Wu, Caide Lu

**Affiliations:** Department of Hepatopancreatobiliary Surgery, Ningbo Medical Center Lihuili Hospital, Ningbo University, Ningbo, Zhejiang 315040 China

**Keywords:** Perihilar cholangiocarcinoma, Lymph node metastasis, Nomogram, Prediction model

## Abstract

**Backgroud:**

We aimed to develop a novel preoperative nomogram to predict lymph node metastasis (LNM) in perihilar cholangiocarcinoma (pCCA) patients.

**Methods:**

160 pCCA patients were enrolled at Lihuili Hospital from July 2006 to May 2022. A novel nomogram model was established to predict LNM in pCCA patients based on the independent predictive factors selected by the multivariate logistic regression model. The precision of the nomogram model was evaluated through internal and external validation with calibration curve statistics and the concordance index (C-index). Receiver operating characteristic (ROC) curve and decision curve analysis (DCA) were used to evaluate and determine the clinical utility of the nomogram.

**Results:**

Multivariate logistic regression demonstrated that age (*OR* = 0.963, 95% CI: 0.930–0.996, *P* = 0.030), CA19-9 level (> 559.8 U/mL vs. ≤559.8 U/mL: *OR* = 3.162, 95% CI: 1.519–6.582, *P* = 0.002) and tumour diameter (*OR* = 1.388, 95% CI: 1.083–1.778, *P* = 0.010) were independent predictive factors of LNM in pCCA patients. The C-index was 0.763 (95% CI: 0.667–0.860) and 0.677 (95% CI: 0.580–0.773) in training cohort and validation cohort, respectively. ROC curve analysis indicated the comparative stability and adequate discriminative ability of nomogram. The sensitivity and specificity were 0.820 and 0.652 in training cohort and 0.704 and 0.649 in validation cohort, respectively. DCA revealed that the nomogram model could augment net benefits in the prediction of LNM in pCCA patients.

**Conclusions:**

The novel prediction model is useful for predicting LNM in pCCA patients and showed adequate discriminative ability and high predictive accuracy.

## Background

Perihilar cholangiocarcinoma (pCCA) is a highly malignant and metastatic disease that represents approximately 50–60% of cholangiocarcinomas (CCAs) [1], and its incidence has increased dramatically worldwide in recent years. pCCA arises between the second-order bile ducts and the insertion of the cystic duct onto the common bile duct [2], as first described by Klatskin in 1965 [3]. Currently, surgical resection is the main potential cure for pCCA, but less than 25% of patients present with treatable early-stage disease [4], and the overall survival (OS) rate is extremely poor [5, 6]. Lymph node metastasis (LNM) has been reported as a significant prognostic indicator of OS for patients with pCCA after surgical resection [7, 8]. The 5-year survival probability of pCCAs with node-positive disease is less than 25% [9, 10]. Some studies have reported that more than 30% of patients with pCCA who undergo curative resection experience LNM [11–13]. Kobayashi et al. reported that the 3-year recurrence rate of pCCA was 80% in patients with LNM [14]. pCCA recurrence in patients with LNM continues to rise and is approximately 100% with adequate follow-up [15]. Aoba and Giuliante recommended obtaining more than 5 regional lymph nodes which may decrease the false negative rate and be beneficial for accurate pCCA staging [16, 17]. Lymph node dissection can contribute to making accurate prognosis judgements and reduce the risk of local tumour recurrence. Local recurrence of pCCA after surgical resection provides an important justification for the use of adjuvant therapy [18]. Some studies reported that pCCA patients with LNM were more likely to improve survival benefits when receiving adjuvant chemotherapy or chemoradiation [19–21]. Parente A et al. indicated that neoadjuvant chemotherapy can increase the OS for pCCA, no matter LNM negative or positive disease [22]. Kuriyama N et al. found that neoadjuvant chemotherapy was feasible and tolerable based on resectability classification and lymph node status [23]. Therefore, accurate preoperative assessment and prediction of LNM is beneficial for guiding the development of surgical treatment strategies for patients with pCCA.

Precise assessment of regional lymph node involvement and the detection of distant metastases are extremely important when deciding upon the appropriate surgical treatment option for patients with pCCA. Currently, imaging examinations including conventional chest radiography, computed tomography (CT), magnetic resonance imaging (MRI), radionuclide scintigraphy, and positron emission tomography (PET) are still the main means of preoperative evaluation for patients with pCCA. PET has been proven to be the most effective method for the detection and characterization of tumour metastasis and has greater sensitivity and specificity than other imaging examinations [24, 25]. Although PET is currently used as an advanced method for detecting tumour metastasis, the sensitivity in the diagnosis of LNM in CCAs is not ideal. Li J et al. found that the sensitivity and specificity of PET were 41.7% and 80%, respectively, in detecting LNM and distant metastasis in pCCA [25]. A meta-analysis indicated that the sensitivity of PET was only 0.520 but had a high specificity of 0.920 in the detection of N stage, while the diagnosis of LNM in CCA using 18-fludeoxyglucose PET is still limited [26]. Therefore, there is an urgent need for clinicians to evaluate LNM and distant metastasis accurately in pCCA before curative surgery.

In current, neoadjuvant chemotherapy exhibits the potential benefits for pCCA patients. Neoadjuvant chemotherapy and radiotherapy are needed to improve the prognosis of pCCA patients with LNM because of poor efficacy of a surgical treatment alone. In addition, the prediction of LNM will benefit from personalized lymph node dissection in pCCA patients. In the present study, we focused on constructing a new nomogram model to predict LNM in pCCA patients using easily collected preoperative clinical data. We expect this predictive tool to be beneficial for the individualized detection of LNM by improving sensitivity substantially, especially when combined with PET/MRI/CT scans.

## Materials and methods

### Study design and patients

**Fig. 1:**
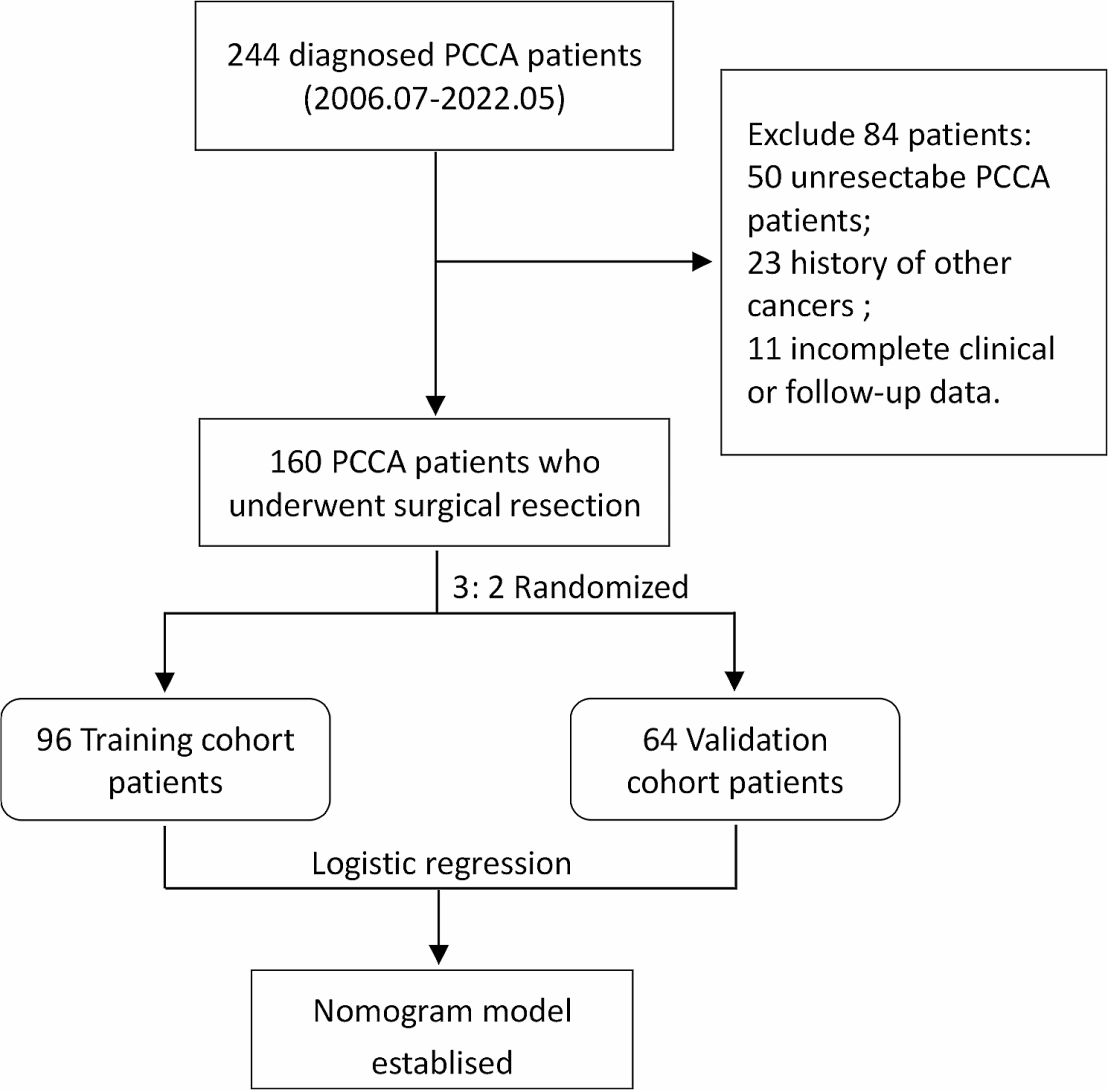
The flowchart of the enrolled pCCA patients

A total of 160 pathologically diagnosed pCCA patients who underwent surgical resection were enrolled at the Ningbo Medical Center Lihuili Hospital from July 2006 to May 2022. Ninety-six pCCA patients were randomly selected for the training cohort, and sixty-four patients were selected for the validation cohort, as shown in Fig. 1. The inclusion criteria of this study included (1) pathologically diagnosed pCCA patients and (2) patients who underwent surgical resection. The exclusion criteria of this study included (1) perioperative death; (2) history of other cancers; and (3) incomplete clinical data.

### Ethics approval

This study was reviewed and approved by the ethics committee of our hospital (Approval number: KY2021PJ146/061). We confirmed that this study was conducted following the Declaration of Helsinki.

### Clinical characteristics

The baseline clinical data of pCCA patients were collected from our hospital information system (HIS). The demographics and clinical and pathologic examination results were recorded for each pCCA patient. Clinical characteristics included age, sex, Bismuth‒Corlette classification, preoperative carcinoembryonic antigen (CEA) level, preoperative carbohydrate antigen 19-9 (CA19-9) level, preoperative direct bilirubin (DBIL), preoperative total bilirubin (TBIL), maximum tumour diameter of preoperative imaging examination, calculi, jaundice, pathological differentiation, cN stage, number of lymph node dissection, T stage, and resection margins.

### Statistical analysis

Measurement and counting data are described as mean (standard deviation) and counts (percentages), respectively. Student’s t test and the chi-squared test were used to assess the differences between groups. The predictive factors of LNM included in the nomogram were identified by univariate and multivariate logistic regression. Receiver operating characteristic (ROC) curve and decision curve analysis (DCA) were performed to determine the diagnosability and net benefit of the nomogram for clinicians in practice.The threshold for tumor biomarker levels is determined through X-tile software based on their value in the prognosis of pCCA patients. SPSS 25.0 (IBM Corporation, 2020, USA) and R version 4.2.2 were used in our present study. *P* < 0.05 was considered to reflect statistical significance.

## Results

### Baseline characteristics

**Fig. 2:**
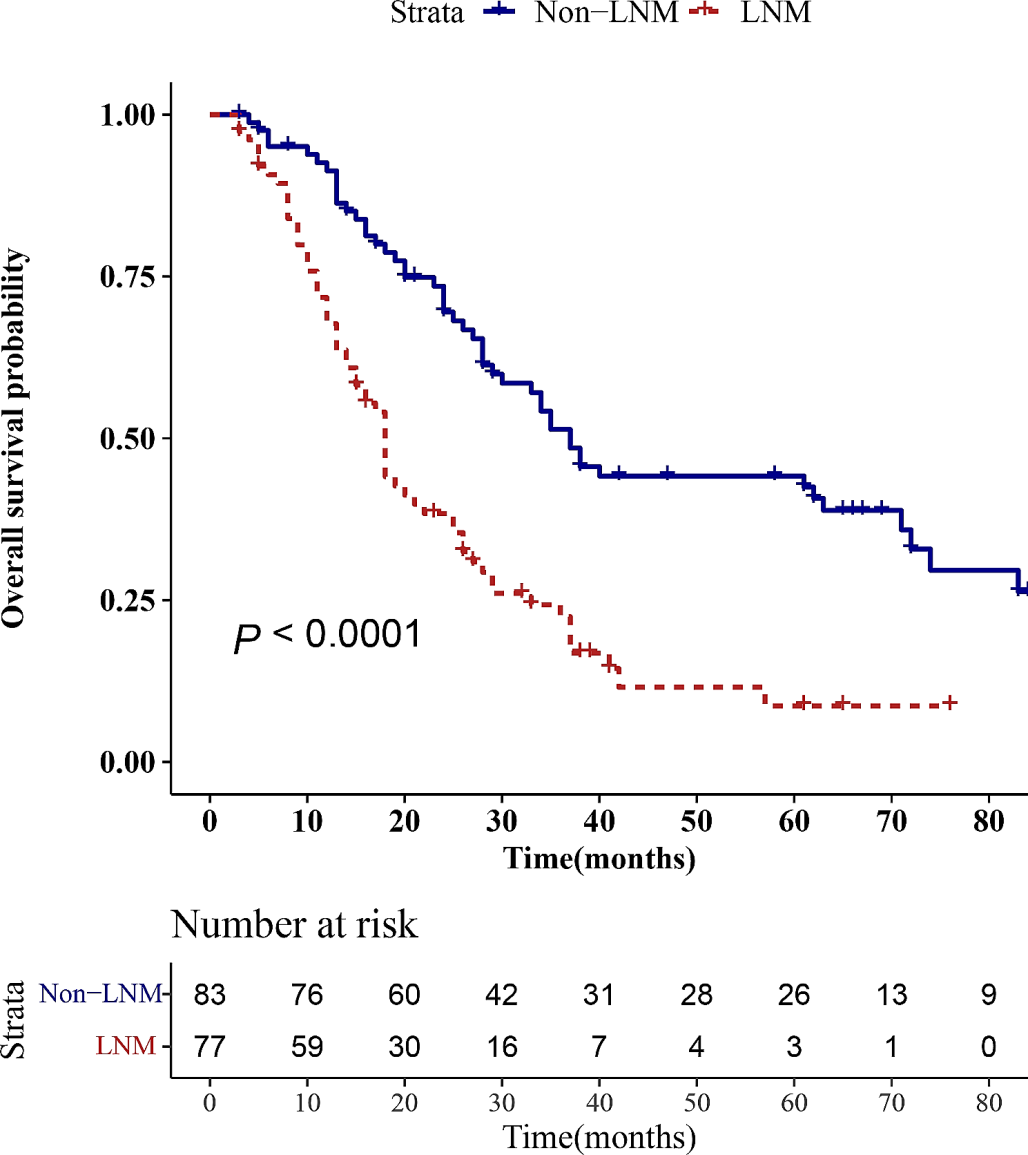
Survival analysis of pCCA patients with lymph node metastasis

One hundred and sixty pCCA patients (ninety-six in the training cohort and sixty-four in the validation cohort) were included in our present research. Of the 160 patients, 77 (48.1%) patients had LNM (50 (50/96, 52.1%) in the training cohort and 27 (27/64, 42.2%) in the validation cohort) on pathologic examination after surgical resection. The median time of follow-up was 24 (range: 3-150) months, 112 patients died, and 48 patients survived up to the current follow-up time point. There were no significant differences between the training and validation cohorts in any of the characteristics except for age (*P* > 0.05). The analysis of baseline characteristics between the two groups is shown in Table 1. In addition, survival analysis indicated that the 1-, 2-, 3-, and 5-year OS rates were 82.6%, 56.7%, 37.6%, and 28.6%, respectively, across the 160 pCCA patients, and the median survival time was 27 months. In the LNM group, the 1-, 2-, 3-, and 5-year OS rates were 71.8%, 38.4%, 22.4%, and 11.5%, respectively, and the median survival time was 18 months. In the non-LNM group, the 1-, 2-, 3-, and 5-year OS rates were 91.3%, 69.5%, 51.4%, and 44.2%, respectively, and the median survival time was 37 months. The log-rank test indicated that there was a significant difference between the LNM group and the non-LNM group in OS rates (*P* < 0.001), as shown in Fig. 2.

**Table 1:** Clinical and pathological baseline of validation and training cohort

Variables	Validation cohort		Training cohort	χ2/t value	*P*
	Mean ± SD/N (%)		Mean ± SD/N (%)		
Gender(male/female)	39(60.9%)/25(39.1%)		54(56.3%)/42(43.8%)	0.347	0.556
Age,years	66.2 ± 8.9		62.1 ± 10.6	2.488	0.014
Bismuth-Corlette classification(I/II/IIIA/IIIB/IV)	8(12.5%)/6(9.4%)/15(23.4%)/19(29.7%)/16(25.0%)		8(8.3%)/16(16.7%)/16(16.7%)/23(24.0%)/33(34.4%)	4.642	0.326
CEA level, ng/ml(≤ 5/>5)	47(73.4%)/17(26.6%)		69(71.9%)/27(28.1%)	0.047	0.828
CA19-9 level, U/mL(≤ 559.8/>559.8)	47(73.4%)/17(26.6%)		61(63.5%)/35(36.5%)	1.714	0.190
DB level, umol/l (≤ 8/>8)	9(14.1%)/55(85.9%)		24(25.0%)/72(75.0%)	2.806	0.094
TB level,umol/l (≤ 34/>34)	11(17.2%)/53(82.8%)		23/(24.0%)/73(76.0%)	1.052	0.305
Tumor diameter, cm	2.9 ± 1.3		3.3 ± 1.6	1.707	0.090
Biliary calculi(no/yes)	48(75.0%)/16(25.0%)		77(80.2%)/19(19.8%)	0.610	0.435
Pathological differentiation(poor/moderate/well)	27(42.2%)/32(50.0%)/5(7.8%)		36(37.5%)/52(54.2%)/8(8.3%)	0.354	0.838
cN stage(0/N1/N2)	37(57.8%)/22(34.4%)/5(7.8%)		46(47.9%)/40(41.7%)/10(10.4%)	1.530	0.465
Number of lymph node dissection(< 7/≥7)	28(43.8%)/36(56.3%)		32(33.3%)/64(66.7%)	1.778	0.182
AJCC T stage (T1-2 / T3-4)	29(45.3%)/35(54.7%)		32(33.3%)/64(66.7%)	2.336	0.126
Resection margins(R0/R1)	58(90.6%)/6(9.4%)		76(79.2%)/20(20.8%)	3.705	0.054

### Predictive factor analysis for LNM and nomogram model construction

**Fig. 3:**
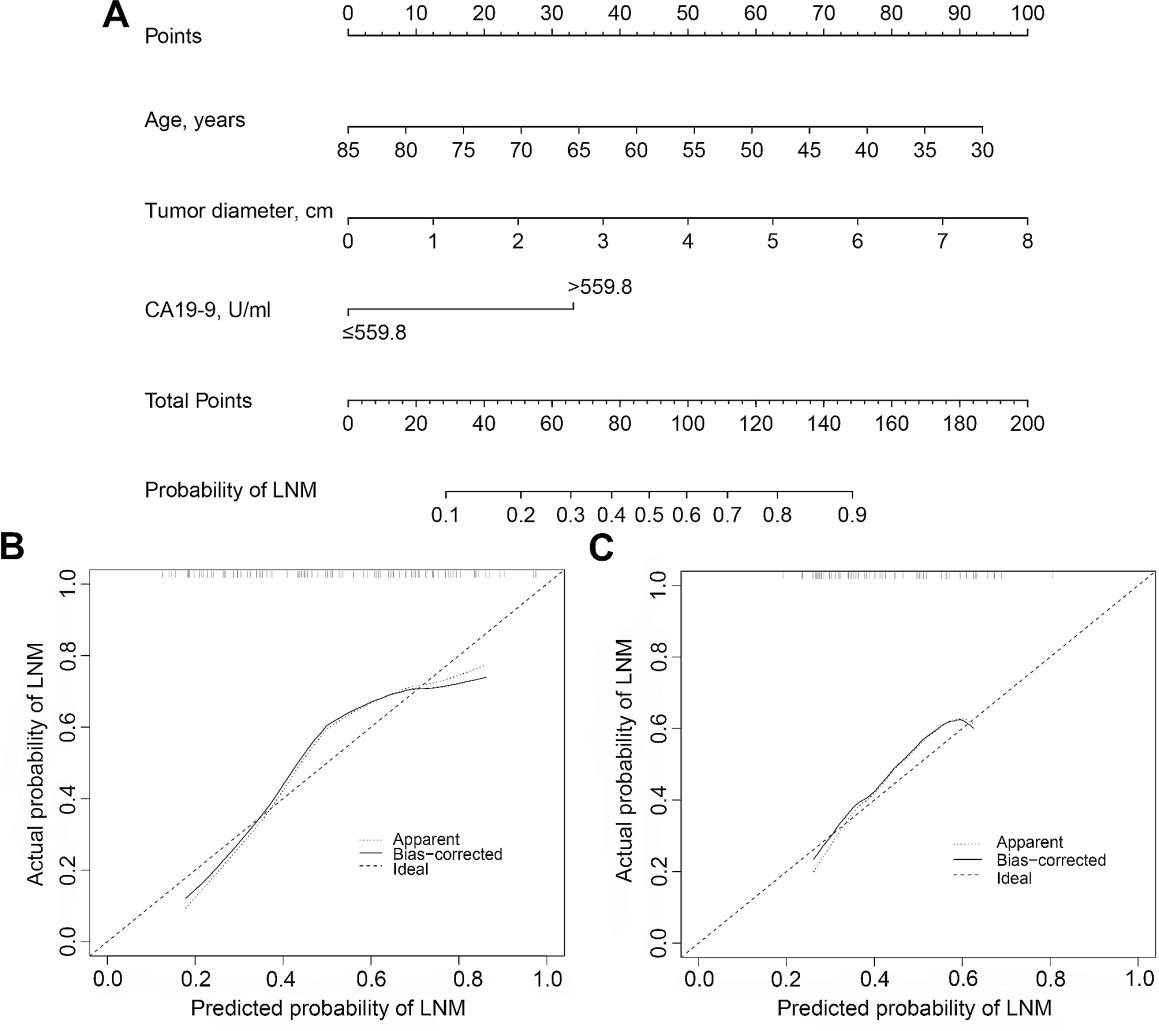
Developed preoperative nomogram clinical model for predicting lymph node metastasis in pCCA patients. **(A)** Nomogram prediction model. (**B**) Calibration curve plots of the training cohort; **(C)** Calibration curve plots of the valitation cohort

Multivariate logistic regression indicated that age (*OR* = 0.963, 95% CI: 0.930–0.996, *P* = 0.030), CA19-9 level (> 559.8 vs. ≤559.8, *OR* = 3.162, 95% CI: 1.519–6.582, *P* = 0.002), and tumour diameter (*OR* = 1.388, 95% CI: 1.083–1.778, *P* = 0.010) were independent predictive factors of LNM in pCCA patients (Table 2). Finally, the nomogram model was constructed incorporating the variables of age, CA19-9, and tumour diameter (Fig. 3A). The nomogram model achieved good concordance, with a C-index of 0.763 (95% CI: 0.667–0.860) and 0.677 (95% CI: 0.580–0.773) in the training cohort and validation cohort, respectively. The calibration curves showed good agreement between the predicted and actual probabilities of LNM in the training and validation cohorts, and the standard lines largely overlapped (Fig. 3B-C).

**Table 2:** Univariate and multivariate logistic regression analysis of LNM in pCCA patients

Variables	Univariate			Multivariate	
	*P*	OR (95%CI)		*P*	OR (95%CI)
Age	0.026	0.964(0.933–0.996)		0.030	0.963(0.930–0.996)
CA19-9 level, U/mL(> 559.8 VS ≤ 559.8)	< 0.001	3.677(1.816–7.446)		0.002	3.162(1.519–6.582)
Tumor diameter	0.003	1.447(1.139–1.839)		0.010	1.388(1.083–1.778)

### Receiver operating characteristic curve and decision curve analysis of the nomogram model

**Fig. 4:**
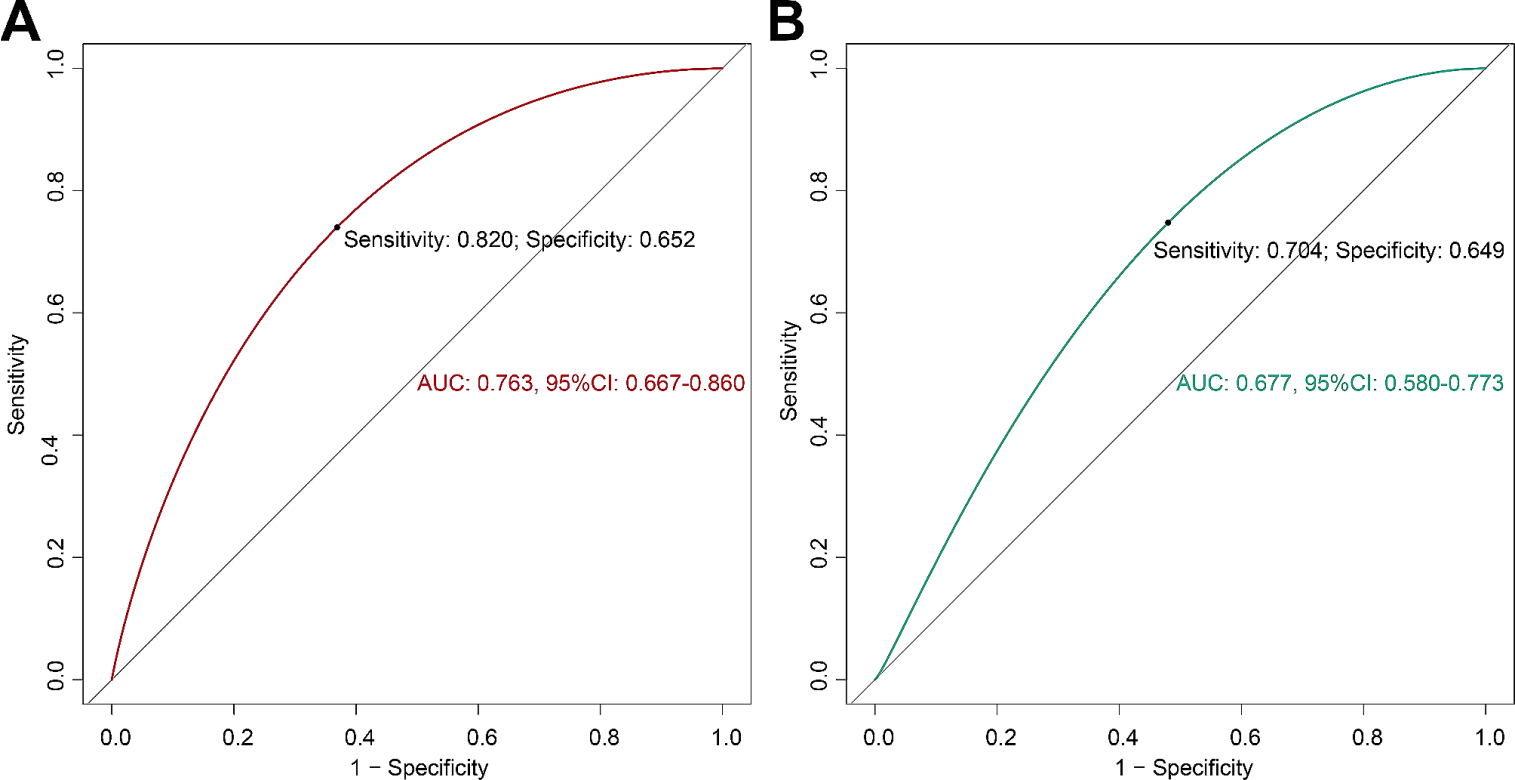
Receiver operating characteristic curve analysis of nomogram model for lymph node metastasis. **(A)** The ROC of training cohort; **(B)** The ROC of valitation cohort

**Fig. 5:**
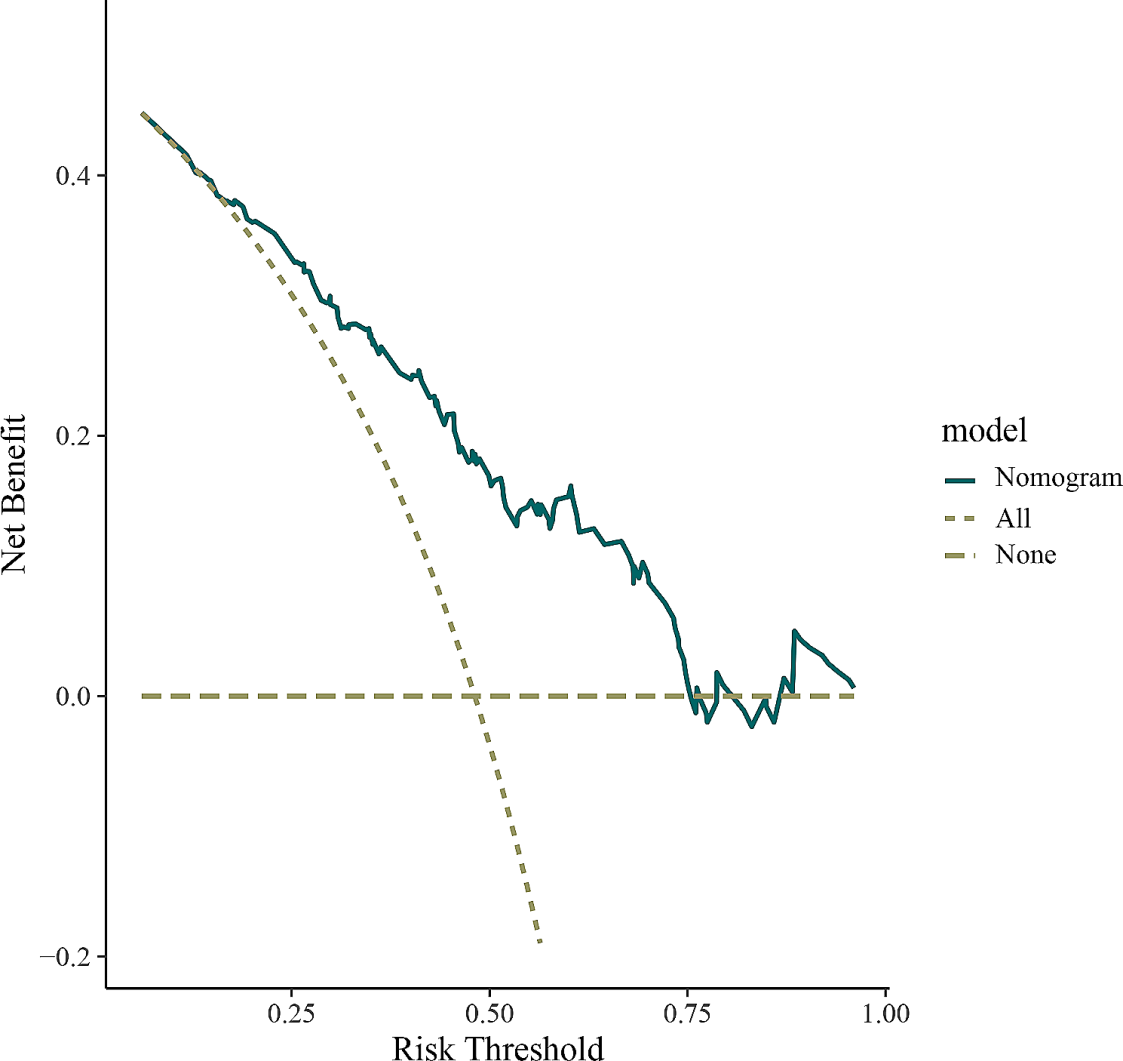
Decision curve analysis of developed nomogram model

ROC curve analysis indicated the comparative stability and adequate discriminative ability of the model (Fig. 4). The area under the ROC curve (AUC) was consistent with the C-index. The sensitivity and specificity were 0.820 and 0.652 in the training cohort (Fig. 4A) and 0.704 and 0.649 in the validation cohort, respectively (Fig. 4B). DCA revealed that the nomogram could augment net benefits and exhibited a wider range of threshold probabilities in the prediction of LNM in pCCA patients (Fig. 5).

## Discussion

In the present research, we constructed a novel nomogram to predict LNM in pCCA patients using preoperative clinical indicators. In addition, the C-index and AUC were 0.763 and 0.677 in the training cohort and validation cohort, respectively, and the sensitivity of the ROC curve was 0.820 in the training cohort and 0.704 in the validation cohort. The predicted probability of LNM was approximately 82% when age = 50, tumour diameter = 3 cm, and CA19-9 level > 559.8 U/ml, and the corresponding total score of the nomogram was approximately 131.5. The nomogram model demonstrates sufficient discriminability and ideal prediction capability for LNM.

As the most common type of CCA, the incidence of pCCA has increased annually worldwide. Some studies have reported that more than 30% of patients with pCCA who undergo curative resection have LNM [11–13]. LNM has been reported as a significant prognostic indicator of OS for patients with pCCA after surgical resection [7, 8]. In the present study, the incidence of LNM was 48.1%, and age, CA19-9, and tumour diameter can be used as predictive factors for LNM in pCCA. In breast cancer, Faleh S et al [27] indicated that the proportion of axillary lymph node metastasis increases with younger age at diagnosis, and the highest proportion occurs when the age is less than 40. A SEER data-based study found that age ≤ 70 was an independent risk factor for LNM in gastric cancer [28]. In our study, we first found that lower age was also an independent risk factor for LNM in pCCA. CA19-9 has become a common diagnostic tumour glycobiomarker for CCAs, but it demonstrates limited diagnosability because it is generally not elevated in early CCA stages. Consequently, the combination of CA19-9 and other biomarkers is often used for auxiliary diagnosis and prognostic evaluation in CCAs [29, 30]. Wang et al [31] found that elevated CA19-9 levels (> 37 U/ml) could be a clinical predictor for regional lymph node staging in pCCA, which is consistent with the findings in the present study. However, the cut-off value of CA19-9 for the prediction of LNM was 559.8 U/ml in our study. However, the CA19-9 level (≥ 1000 U/ml) did not show a significant predictive effect for lymph node metastasis in biliary tract cancer (BTC)-unclassified cholangiocarcinomas either in the radiomics model or clinical model [32]. Further research on the optimal threshold CA19-9 value in the prediction of LNM is needed. Zhang et al [33] noticed that the probability and number of metastatic lymph nodes increase with larger tumour size in pCCA. The clinical model constructed by Ji GW et al [32] also indicated that CT-reported tumour size was a risk factor for LNM in BTC-unclassified cholangiocarcinomas. Tumour size is an important risk factor for LNM and has also been observed in gastric cancer, breast cancer, thyroid cancer, and other cancers [34–36].

The 8th edition of the American Joint Committee on Cancer Tumor, Nodes, Metastases recommended obtaining at least 5 regional lymph nodes in pCCA surgery [2]. Aoba and Giuliante recommended obtaining more than 5 regional lymph nodes which may decrease the false negative rate and be beneficial for accurate pCCA staging [16, 17]. Lymph node dissection can contribute to making accurate prognosis judgements and reduce the risk of local tumour recurrence. Therefore, accurate preoperative assessment of LNM is beneficial for guiding the development of surgical treatment strategies for patients with pCCA. Currently, some nomogram models have been reported to predict LNM in intrahepatic cholangiocarcinoma (iCCA) because of the limited, low sensitivity of imaging for LNM in CCAs [37, 38], but there are currently few articles on predicting lymph node metastasis in pCCA. In 179 pCCA patients, Wang et al [31] reported an established nomogram model to preoperatively evaluate LNM using a deep learning radiomics signature (DLR), CA19-9 level, CEA level, and CT-reported lymph node staging. The AUCs of the LNM status classifier reached 0.866 in the training cohort and 0.870 in the external cohorts. In our study, the AUCs of the constructed preoperative nomogram clinical model were 0.763 in the training cohort and 0.677 in the validation cohort. Imaging data were not applied and included in our prediction model, which is also one of the limitations of our research. Consequently, multicentre and large-sample studies are still needed.

Several other limitations existed in this study. First, the reported nomogram was established based on retrospective clinical data from a single centre. Multicentre research is needed to validate our prediction model in the future. Second, the possibility of positive LNM also depends on the number of lymph nodes cleaned during surgery and the experience of pathologists, and false negatives may also exist in negative LNM patients. Finally, our study spans 16 years, the surgery had changed and an impact on the judgement of LNM outcome cannot be ignored.

## Conclusion

In this study, we proposed a novel preoperative nomogram clinical model that included age, CA19-9 level, and tumour diameter to facilitate the preoperative evaluation of LNM in patients with pCCA. Our clinical nomogram model showed adequate discriminability and high predictive accuracy and would be beneficial for clinical decision-making and guiding the development of surgical treatment strategies for patients with pCCA.

## Data Availability

The datasets used or analyzed during the current study are available from the corresponding author on reasonable request. In order to protect study participants’ privacy, our data cannot be shared openly.
